# Comparison of computed tomography pulmonary angiography and point-of-care tests for pulmonary thromboembolism diagnosis in dogs

**DOI:** 10.1111/jsap.12185

**Published:** 2014-02-13

**Authors:** R Goggs, D L Chan, L Benigni, C Hirst, L Kellett-Gregory, V L Fuentes

**Affiliations:** Department of Clinical Science and Services, Royal Veterinary CollegeNorth Mymms, AL9 7TA

## Abstract

**Objectives:**

**To evaluate the feasibility of CT pulmonary angiography for identification of naturally occurring pulmonary thromboembolism in dogs using predefined diagnostic criteria and to assess the ability of echocardiography, cardiac troponins, D-dimers and kaolin-activated thromboelastography to predict the presence of pulmonary thromboembolism in dogs.**

**Methods:**

**Twelve dogs with immune-mediated haemolytic anaemia and evidence of respiratory distress were prospectively evaluated. Dogs were sedated immediately before CT pulmonary angiography using intravenous butorphanol. Spiral CT pulmonary angiography was performed with a 16 detector-row CT scanner using a pressure injector to infuse contrast media through peripheral intravenous catheters. Pulmonary thromboembolism was diagnosed using predefined criteria. Contemporaneous tests included echocardiography, arterial blood gas analysis, kaolin-activated thromboelastography, D-dimers and cardiac troponins.**

**Results:**

**Based on predefined criteria, four dogs were classified as pulmonary thromboembolism positive, three dogs were suspected to have pulmonary thromboembolism and the remaining five dogs had negative scans. The four dogs identified with pulmonary thromboembolism all had discrete filling defects in main or lobar pulmonary arteries. None of the contemporaneous tests was discriminant for pulmonary thromboembolism diagnosis, although the small sample size was limiting.**

**Clinical Significance:**

**CT pulmonary angiography can be successfully performed in dogs under sedation, even in at-risk patients with respiratory distress and can both confirm and rule out pulmonary thromboembolism in dogs.**

## INTRODUCTION

Pulmonary thromboembolism (PTE) is the obstruction of the pulmonary artery or its branches by thrombi and is a major cause of morbidity and mortality in dogs with immune-mediated haemolytic anaemia (IMHA) (Reimer *et al*. 1999, Scott-Moncrieff *et al*. 2001). Dogs with IMHA are predisposed to PTE potentially because of an associated hypercoagulable state (Fenty *et al*. 2011, Goggs *et al*. 2012) that may result from increased intravascular tissue factor expression secondary to the marked inflammatory response that accompanies the disease (Piek *et al*. 2011, Kidd & Mackman 2013). There is also evidence of platelet activation in dogs with IMHA (Weiss & Brazzell 2006). As such, both anticoagulants and antiplatelet agents are used to reduce PTE risk and manage thrombotic complications, but are often empirically prescribed due to difficulties with definitively identifying PTE antemortem (Goggs *et al*. 2009).

In humans, rapid, multi-slice spiral computed tomography pulmonary angiography (CTPA) is central to PTE diagnosis (Fesmire *et al*. 2011). CTPA studies are obtained by simultaneous thoracic CT scanning and bolus injection of contrast media. Iodinated contrast agents are rapidly infused through peripheral catheters using a pressure injector, linked electronically to the scanner to maximally enhance the pulmonary arteries (Habing *et al*. 2011). Occlusive or partial filling defects in pulmonary arteries are diagnostic for PTE (Wittram *et al*. 2004), while a normal CTPA study helps to rule out PTE as the cause of respiratory distress, unless the index of suspicion is very high (Torbicki *et al*. 2008). Multi-detector-row or multi-slice CT scanners are increasingly available in veterinary medicine and permit imaging of the whole thorax without the need for general anaesthesia. In dogs, multi-slice CT angiography has been used for experimental PTE studies (Takahashi *et al*. 2008), and to identify a descending aortic thrombus in a dog with spirocercosis (Kirberger & Zambelli 2007). CTPA has been used to investigate the incidence of PTE following non-cemented total hip arthroplasty in dogs, although no evidence of PTE was identified (Tidwell *et al*. 2007). CTPA has been successfully used to diagnose pulmonary embolism in dogs with naturally occurring heartworm disease (Jung *et al*. 2010).

Although in humans CTPA is the definitive test for PTE, other diagnostics are used to determine which patients require advanced imaging and for severity scoring to guide management. In humans presenting with compatible clinical signs but no prior thrombotic history, rapid quantitative D-dimer assays are performed prior to imaging (Wells *et al*. 2000, Huisman & Klok 2013). Where PTE is present, echocardiography and cardiac biomarkers including cardiac troponins can quantify the haemodynamic consequences of PTE and provide prognostic information. In humans with PTE, serum cardiac troponin values are highly correlated with cardiac myocyte injury, degree of cardiac dysfunction and with prognosis (Janata *et al*. 2003, Becattini *et al*. 2007). Typical echocardiographic findings in dogs with PTE include right ventricular (RV) dilatation, pulmonary arterial hypertension and paradoxical septal motion (Venco *et al*. 1998, Johnson *et al*. 1999). In humans with severe PTE, the RV apex is spared the hypokinesis that affects the remainder of the free wall. This apical sparing (the McConnell sign) is highly specific for PTE in humans, but has not been reported in dogs (McConnell *et al*. 1996). More recently, echocardiographic measurement of the tricuspid annular plane systolic excursion (TAPSE) and right ventricular myocardial performance index (TEI index) have been reported to be of value in humans with acute PTE (Park *et al*. 2008, Rydman *et al*. 2010, Park *et al*. 2012). To date, studies of D-dimers, cardiac troponins and echocardiography have not been carried out in dogs with PTE but might improve the initial assessment of PTE risk and provide prognostic information in known PTE cases.

The aims of this pilot study were therefore to evaluate the feasibility of CTPA for identification of naturally occurring PTE in dogs using predefined diagnostic criteria, and to evaluate the predictive ability of echocardiography, cardiac troponins, D-dimers and kaolin-activated thromboelastography (TEG) for canine PTE diagnosis.

## MATERIALS AND METHODS

### Sample size

A cohort of 12 dogs with IMHA was predetermined for this pilot study. Previous studies suggest 32 to 80% dogs with IMHA have postmortem evidence of PTE (Klein *et al*. 1989, Carr *et al*. 2002). It was therefore estimated that 4 to 10 dogs with IMHA and evidence of respiratory distress would have PTE detectable by CTPA and that this would provide sufficient positive and negative cases to evaluate the feasibility of CTPA for diagnosis of canine PTE.

### Animals

Twelve client-owned dogs diagnosed with IMHA admitted to the Queen Mother Hospital for Animals, The Royal Veterinary College between November 2009 and January 2013 were prospectively evaluated. Written, informed owner consent was obtained at hospital admission. The diagnosis of IMHA was based on the presence of regenerative anaemia and at least one of the following: positive in-saline agglutination test, positive direct antibody (Coombs’) test or moderate-marked spherocytosis identified on peripheral blood smear examination by a board-certified clinical pathologist. Additional inclusion criteria were the combination of either tachypnoea (respiratory rate >20/min) or PaCO_2_ < 32 mmHg, plus either increased respiratory effort or hypoxaemia (SaO_2_ < 95% or PaO_2_ < 85 mmHg on room air or a PaO_2_:FiO_2_ ratio of <400 mmHg on supplemental oxygen). Cases were ineligible if there was concurrent thrombocytopenia (<100 × 10^3^/μL) or bodyweight <7 · 5 kg. Signalment, previous medical history and physical examination findings at admission were recorded.

### Ethics statement

This study was approved by the institutional ethics and welfare committee (Ref: 20111133R). Case management was determined by the primary attending clinicians. To minimise risks associated with sedation, all cases were stabilised as appropriate prior to CTPA and flow-by oxygen provided during the procedure. To minimise risks associated with contrast material administration, dogs with known hypersensitivity to iodinated contrast agents were excluded. Fluid or electrolyte disorders were corrected prior to CTPA. In cases with renal insufficiency, fluid diuresis with physiologic saline following CTPA was at the primary clinician's discretion.

### CTPA

Dogs were sedated immediately before CTPA using a dose of 0 · 3 mg/kg butorphanol (Torbugesic 1%; Zoetis) intravenously and positioned in sternal recumbency. Spiral CT pulmonary angiography was performed using a 16-slice CT scanner (Mx8000 IDT; Philips Healthcare). Initial precontrast survey CT scans of the thorax were performed with scan time <30 seconds. Boluses of 2 mL/kg (600 mg/kg) of 300 mg I/mL iohexol (Omnipaque 300; GE Healthcare) were then administered via peripheral intravenous catheters at 2 to 3 mL/s dependent on bodyweight using a pressure injector (Stellant; Medrad) with a maximum injection pressure of 150 psi. CT images were acquired immediately after the beginning of the contrast injection in order to capture the pulmonary artery phase. CT scan parameters were 120 kV, 150 to 250 mAs (dependent on patient size), 3 mm slice thickness, 1 · 5 mm increment, 0 · 688 pitch and sharp filter applied.

### Criteria for PTE diagnosis

The CTPA studies were reported by board-certified radiologists at the time of the scans. Images were subsequently blindly reviewed by two observers (LB, VLF) and predefined criteria applied for PTE diagnosis (Table 1). Initial imaging reports were then reviewed, any discrepancies evaluated and consensus on diagnoses reached.

**Table 1:** A summary of the criteria used for diagnosis of pulmonary thromboembolism (PTE) by computed tomography pulmonary angiography (CTPA) in this study

PTE diagnosis	Criteria (only one required per category)
Positive	Complete pulmonary arterial occlusion.
	Central intraluminal arterial filling defect(s) present.
	Peripheral intraluminal arterial filling defect(s) present.
Suspicious	Luminal irregularities in central or peripheral pulmonary arteries.
	Differences in contralateral arterial luminal density.
	Multi-focal alveolar pattern with no probable alternative diagnosis.
Negative	None of the above.

Only one criterion in the positive category was necessary for that classification to be assigned. If none of these criteria were satisfied, then any one of the criteria in the *suspicious* category led to classification of the patient as *suspicious* for PTE. A *negative* diagnosis was only made when none of the criteria listed were satisfied

### Echocardiography

Echocardiographic examinations were performed by board-certified cardiologists or cardiology residents directly supervised by board-certified cardiologists, using a dedicated cardiac ultrasound machine (Vivid 7; GE Healthcare). All scans were subsequently reviewed (VLF) and a judgement was made regarding the presence or absence of the McConnell sign (apical sparing of right ventricular hypokinesis). Pulmonary artery (PA) pressures were estimated using spectral Doppler echocardiographic blood flow velocities of tricuspid and pulmonic insufficiency. The right ventricular Tei index and the tricuspid annular plane systolic excursion (TAPSE) normalised to aortic diameter were calculated as previously reported (Teshima *et al*. 2006, Pariaut *et al*. 2012).

### Blood sampling and clinical pathology

Blood samples were collected by jugular venepuncture using 21 G needles and appropriately sized syringes and immediately aliquoted into 1 · 1 or 1 · 3 mL non-vacuum, polypropylene tubes (Paediatric tube; International Scientific Supplies). Samples for complete blood counts (CBC) were collected into potassium EDTA tubes. Samples for serum biochemistry were collected into gel separator tubes. For TEG and D-dimer determinations blood was collected into 3 · 2% liquid sodium citrate tubes. CBCs and serum biochemistry were performed using automated analysers (Cell-Dyn 2500; Abbott Laboratories and ILAB 600; Instrumentation Laboratory) and the values were cross-checked by board-certified clinical pathologists. Samples for arterial blood gas analysis were collected by direct arterial puncture into non-vacuum heparinised syringes (Preset syringe; BD) and measured immediately with a point-of-care analyser (Stat-Profile 9; Nova Biomedical). During normal working hours, plasma D-dimers were analysed using a latex agglutination method (Helena Biosciences). Outside normal hours, plasma D-dimers were assayed using a quantitative point-of-care assay (Nycocard D-dimer; Axis-Shield POC) validated for use in dogs (Dewhurst *et al*. 2008). Cardiac troponin I (cTnI) concentrations were measured in serum using an automated immunoassay method previously validated in dogs (Spratt *et al*. 2005).

### Thromboelastography

To avoid time-dependent alterations in TEG parameters, samples were held at room temperature (∼20°C) for 30 minutes before analysis (Wiinberg *et al*. 2005). Kaolin-activated TEG assays were run according to the manufacturer's instructions (TEG 5000; Haemoscope Corporation). Briefly, 1000 μL of citrated whole blood was added to proprietary kaolin vials prewarmed to room temperature and gently mixed by inversion. From this blood:kaolin mix, 340 μL were pipetted into a reaction cup containing 20 μL of 0 · 2M CaCl_2_ prewarmed to 37°C. The reaction cup was raised to the pin and the tracing started. Assays were run for 90 minutes. Heparinase-treated reaction cups were used for dogs with indwelling IV catheters and for those receiving therapeutic heparin, otherwise plain reaction cups were used. Four variables were recorded from TEG tracings: R, K, alpha and MA and compared against method-specific reference intervals generated from residual blood collected for annual health screening of dogs from the hospital blood donation programme.

### Statistical analyses

Data were collated using Excel 2010 (Microsoft) and analysed using Prism 5.0 (GraphPad Software).

## RESULTS

**FIG 1:**
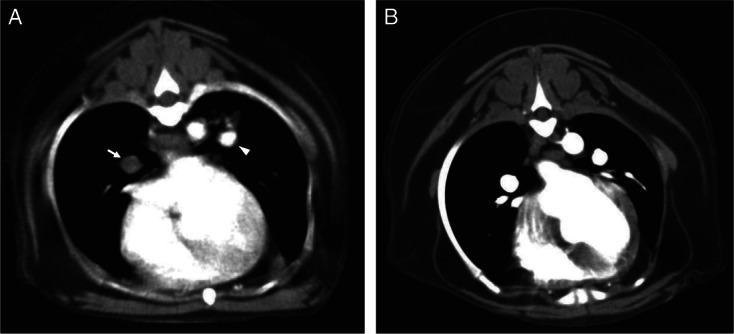
CT pulmonary angiography (CTPA) from two dogs with immune-mediated haemolytic anaemia. (A) Positive CTPA study diagnostic for PTE. Intraluminal filling defects can be clearly seen in both the right (arrow) and left (arrowhead) main pulmonary arteries. The filling defect in the left pulmonary artery is only partial at this level. (B) Negative CTPA study which rules out PTE in this patient. There is normal opacification of both left at right pulmonary arteries by contrast at this level. No aortic filling defects were noted in this study

Based on the predefined CTPA diagnostic criteria, four (33%) dogs were classified as positive for PTE, three (24%) dogs were suspected to have PTE and the remaining five (42%) dogs had negative CTPA scans. The four dogs with PTE definitively identified by CTPA all had discrete filling defects in main or lobar pulmonary arteries (Fig 1). Abnormalities present in the three dogs with scans classified as suspect included possible filling defects in smaller caudal lobe arteries (n = 2), arterial size irregularities (n = 1) and multi-focal alveolar infiltrates consistent with thromboembolic disease (n = 2). Of the five dogs with negative scans, one dog had evidence of cardiac enlargement and another dog had evidence of atelectasis. In the three other dogs with negative CTPA scans an alternative diagnosis to explain the dog's respiratory distress was not identified.

Ten dogs underwent echocardiography; nine had evidence of pulmonic regurgitation and six had tricuspid regurgitation. TAPSE values and Tei indices were calculable in most patients that underwent echocardiography. A subjective assessment of right ventricular wall motion was made in all patients that underwent echocardiography. Apical sparing of right ventricular hypokinesis was subjectively identified in three patients, but this finding did not correlate with CTPA diagnosis.

**FIG 2:**
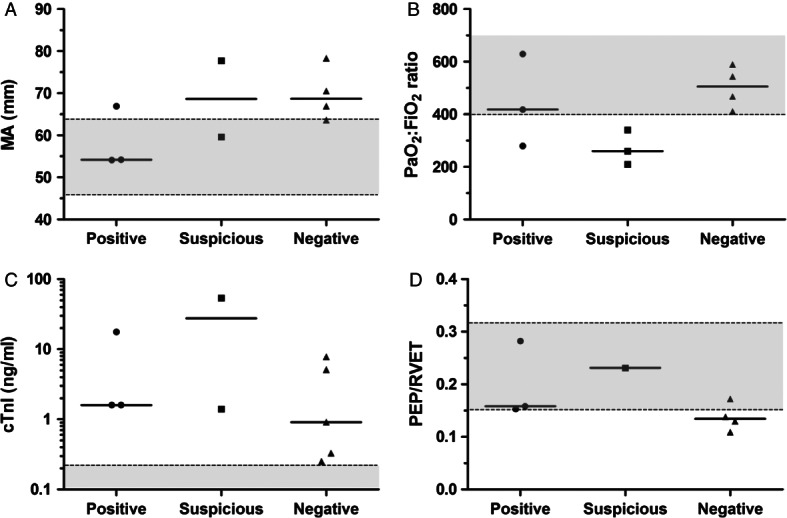
Scatterplots of clinicopathologic and cardiopulmonary parameters stratified by CT pulmonary angiography (CTPA) diagnosis including (A) Kaolin-activation thromboelastography maximum amplitude; (B) PaO_2_:FiO_2_ ratio from arterial blood gas analyses; (C) cardiac troponin I values and (D) pre-ejection period/right ventricular ejection time (PEP/RVET) values. Solid horizontal lines represent the median value. Grey shaded areas between dotted lines represent normal reference intervals

**FIG 3:**
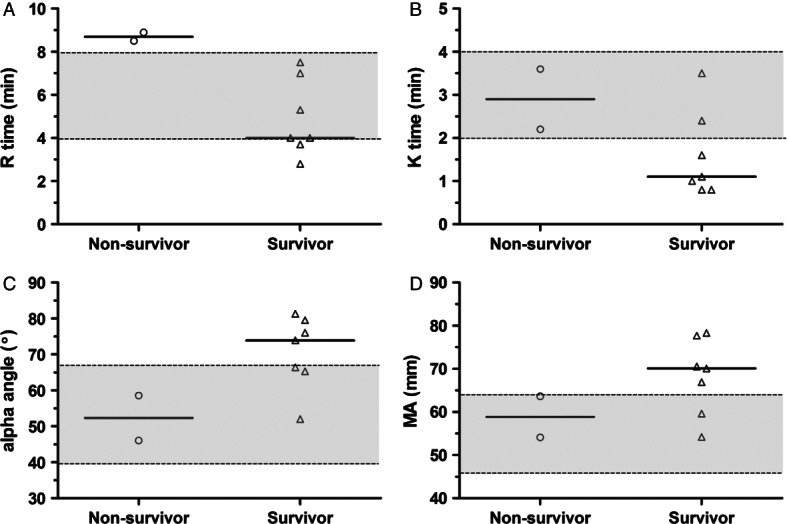
Scatterplots of the four principle thromboelastography variables, reaction time (R), clot formation time (K), clot formation angle (alpha) and maximum amplitude (MA) stratified by outcome. Solid horizontal lines represent the median value. Grey shaded areas between dotted lines represent normal reference intervals

Additional clinicopathologic data collected from the 12 dogs is summarised in Table 2. All blood samples were collected within 4 hours of the CTPA scan being performed. Correlations between clinicopathologic variables and CTPA diagnosis were assessed by visual inspection of scatterplots (Fig 2), but no variable was clearly discriminant for CTPA diagnosis. In terms of outcome, seven dogs survived to discharge, three dogs were euthanised and two dogs died. The non-survivors were evenly distributed across the three CTPA diagnosis groups. None of the cardiopulmonary variables (arterial blood-gases, echocardiographic indices, cardiac troponins) assessed were significantly different between the survivors and the non-survivors. Of the coagulation parameters measured, the kaolin-TEG variables were most associated with outcome, although only the R time of non-survivors was significantly longer than in survivors (P = 0 · 0235, unpaired Student's *t* test; Fig 3).

**Table 2:** A summary of the clinicopathologic data from the 12 dogs stratified by computed tomography pulmonary angiography (CTPA) diagnosis

	CTPA PTE Dx	RR (bpm)	pH	PaO_2_ (mmHg)	PaCO_2_ (mmHg)	A-a (mmHg)	PaO_2_:FiO_2_	SaO_2_ (%)	D-dim (ng/ml)	R (min)	K (min)	Alpha (^o^)	MA (mm)	cTnI (ng/ml)	Tei index	Apical sparing	PEP/RVET	PR vel (m/s)	TR vel (m/s)	TAPSE (mm)	TAPSE/Ao	Outcome
Case			7 · 36 to 7 · 47	91 to 118	26 to 41		>400	95 to 100	<250	4 to 8	2 to 4	40 to 67	46 to 64	<0 · 23	<0 · 4*		0 · 154 to 0 · 319†					
8 · 8 y MC Giant schnauzer	+ve	28	7 · 385	58 · 6	27 · 4	55 · 5	279 · 0	88	500 to 1000	8 · 9	3 · 6	46 · 1	54 · 1		0 · 74	No	0 · 282	—	4 · 05	12 · 4	0 · 44	Euthanised
7 · 2 y FS Irish setter	+ve	48	7 · 455	87 · 7	26 · 8	26 · 8	417 · 6	97 · 7		5 · 3	1 · 6	66 · 4	54 · 2	17 · 6	0 · 15	Yes	0 · 158	1 · 67	2 · 9	20 · 0	0 · 88	Discharged
2 y MC Maltese	+ve	P+												1 · 6								Died
8 · 5 y MC GSD	+ve	38	7 · 416	132 · 2	16 · 5	1 · 1	629 · 5	99 · 3		7	2 · 4	65 · 3	70 · 1	1 · 6	0 · 28	No	0 · 153	1 · 7	—	43 · 0	1 · 74	Discharged
5 · 1 y ME Cocker spaniel	Susp.		7 · 414	83 · 9	36 · 3		209 · 8	94 · 6	>2000	4	1 · 1	73 · 9	59 · 6	53 · 8		No		0 · 93	2 · 91	20 · 0	1 · 00	Discharged
2 · 2 y MC Miniature Dachshund	Susp.	48	7 · 393	54 · 5	22 · 4	66 · 3	259 · 5	87 · 6														Died
10 · 8 y FS Cocker spaniel	Susp.	42	7 · 41	71 · 4	17 · 6	54 · 8	340 · 0	94 · 7	1000 to 2000	2 · 8	0 · 8	79 · 5	77 · 7	1 · 4	0 · 2	Yes	0 · 231	1 · 15	3 · 36	15 · 2	0 · 97	Discharged
4 y FS ESS	−ve		7 · 358	217 · 5	27 · 4		543 · 8	99 · 8						0 · 33		No		1 · 02		21 · 9	1 · 15	Euthanised
10 y FS Cairn terrier	−ve	30	7 · 479	123 · 8	19 · 6	1 · 1	589 · 5	96 · 6		7 · 5	3 · 5	52	66 · 9	7 · 8	0 · 1	No	0 · 138	1 · 22	2 · 84	10 · 0	0 · 78	Discharged
10 · 8 y MC Keeshond	−ve	20	7 · 454	86 · 3	21 · 1	33 · 4	411 · 0	96 · 5	500 to 1000	4	1	76	70 · 5	5 · 1	0 · 15	No	0 · 172	1 · 85	—			Discharged
5 · 8 y FS Tibetan spaniel	−ve	16						96*	>2000	3 · 7	0 · 8	81 · 3	78 · 3	0 · 25	0 · 12	No	0 · 130	1 · 38	—	23 · 3	1 · 70	Discharged
5 · 1 y FS Maltese	−ve	72	7 · 406	98 · 2	25 · 5	20 · 2	467 · 6	98	500 to 1000	8 · 5	2 · 2	58 · 6	63 · 6	0 · 91	0 · 25	Yes	0 · 109	1 · 44	4 · 05	11	0 · 61	Euthanised

A-a Alveolar-arterial oxygen difference, SaO_2_ Arterial oxygen saturation, PaCO_2_ Arterial partial pressure of carbon dioxide, PaO_2_ Arterial partial pressure of oxygen, cTnI Cardiac troponin I, K Clot formation time, Alpha Clot formation angle, D-Dim D-dimers, Dx Diagnosis, ESS English springer spaniel, FE Female entire, FS Female spayed, FiO_2_ Fraction of inspired oxygen, MC Male castrated, ME Male entire, MA Maximum amplitude, PEP/RVET Pre-ejection period/right ventricular ejection time, PTE Pulmonary thromboembolism, PR vel Pulmonic regurgitation velocity, R Reaction time, RR Respiratory rate, Susp. Suspicious, TAPSE/Ao TAPSE normalised to aortic diameter, Tei Tei index, TAPSE Tricuspid annular plane systolic excursion, TR vel Tricuspid regurgitation velocity

* Reference interval from (Teshima *et al*. 2006)

† Reference interval from (Baumwart *et al*. 2005)

## DISCUSSION

This study describes the use of CTPA to establish definitive antemortem diagnoses of naturally occurring PTE in dogs with IMHA. Using CTPA, PTE was confirmed in 33% dogs and either confirmed or suspected in 58% of dogs with IMHA and respiratory distress, values consistent with previous postmortem reports of similar populations (Klein *et al*. 1989, Carr *et al*. 2002). These findings support the assertion that PTE is common in these dogs, and that CTPA is useful for confirming the diagnosis.

The present study is based on the premise that CTPA represents the best available technique for the identification of PTE in dogs. CTPA is recommended for diagnosis of massive PTE in humans (Torbicki *et al*. 2008) and for investigation of those with appropriate clinical probability scores. No studies in dogs have yet compared CTPA with more established techniques such as ventilation/perfusion (V/Q) scanning or selective angiography, or sought to incorporate probability assessments into clinical decision making. There are several potential advantages of CTPA over these tests which are less widely available, require more involved radiation management protocols or necessitate invasive pulmonary artery catheter placement. It is not yet clear that the benefits and diagnostic capabilities of CTPA in humans will directly translate to dogs, particularly given the inherently different anatomy and patient size. Work establishing multi-slice CTPA protocols including those for bolus-tracking studies has recently been published, paving the way for greater use of CTPA in dogs (Drees *et al*. 2011, Cassel *et al*. 2013).

Where PTE was suspected rather than confirmed, multiple small emboli may have been present in mainstem vessels or emboli present only in subsegmental vessels impairing diagnostic ability. In humans, the two major causes of indeterminate CTPA scans are motion artefacts and poor contrast enhancement (Jones & Wittram 2005). Both are possible using a sedated CTPA protocol in dogs given that breath holding to minimise motion artefact and to improve lung aeration cannot be achieved. These potential issues must be considered when undertaking and interpreting CTPA scans. Repeat scans or reconstructions with narrower slices might enable definitive identification or exclusion.

The cause of respiratory distress in the dogs with negative CTPA scans is unclear. In humans, multi-slice CTPA has a low false-negative rate (sensitivity 83 to 100%) (Cronin *et al*. 2008). Sensitivity is lower when emboli are confined to subsegmental vessels (Goodman *et al*. 1995), although multi-slice scans have improved detection rates in humans (Ghaye 2007), particularly as slice thickness is reduced (Jung *et al*. 2011). If these three dogs were truly PTE-negative, then non-respiratory causes of tachypnoea including reduced blood oxygen content, metabolic acidosis, pain, anxiety and medications such as glucocorticoids are all plausible causes in dogs with IMHA (Hall & Lee 2009).

Surprisingly, no clinicopathologic variable assessed reliably related to the CTPA diagnosis. For instance, two dogs with definitively identified PTE had a PaO_2_:FiO_2_ ratio above 400 mmHg. Similarly, two dogs without CTPA evidence of PTE had cTnI values above 5 ng/mL (reference value <0 · 23 ng/mL). This may suggest these diagnostic tests are of limited value for PTE diagnosis in dogs, although the small sample size limits the ability to draw definitive conclusions. Each parameter assessed has distinct sensitivity and specificity characteristics and diverse causes of false-positive or false-negative results. For example, oxygenation impairment is related to pulmonary vascular compromise, thus PTE might be clearly visible on CTPA but have limited impact on PaO_2_:FiO_2_ ratio (McIntyre & Sasahara 1971). Myocardial hypoxia or dysfunction can occur in IMHA and might have been responsible for increased cTnI values in dogs with negative CTPA scans (Prosek & Ettinger 2010). Timing of measurement in relation to the PTE event is also important for certain parameters. D-dimers should be measured within 1 to 2 hours of the suspected event because they peak rapidly after PTE and decline to reference values within 24 hours (Ben *et al*. 2007). In contrast, early measurement of cardiac troponins can lead to false-negative findings in PTE (Ferrari *et al*. 2012). This study was based on the premise that CTPA represents the optimal diagnostic method for PTE diagnosis in dogs and therefore compared the performance of other diagnostics to it. This assumption may not be true for all cases, however, which might explain why some dogs with negative CTPA scans had high cTnI values for instance. Further evaluation of both CTPA and the point-of-care tests for identification of PTE in dogs is clearly necessary before firm conclusions about their value in PTE diagnosis can be drawn.

There were five non-survivors in this study. Three dogs were euthanised due to the severity of the underlying disease, failure to respond to therapy and the development of complications including PTE and anuric kidney failure. One dog with suspected PTE suffered respiratory arrest and died but necropsy was not performed in that case. Necropsy was performed in three cases (2 positive for PTE and 1 negative for PTE). In both cases where PTE was diagnosed by CTPA antemortem, PTE was identified postmortem. No PTE was identified in the dog with negative CTPA, which was euthanised due to development of acute kidney injury.

Few of the variables measured were associated with outcome, although this study was not designed to assess outcome in these dogs. None of the cardiopulmonary variables were useful for outcome stratification. The association between the four TEG variables and outcome in this study was consistent with previous studies of IMHA, wherein dogs with “relative hypocoagulability” were less likely to survive than those with hypercoagulable tracings (Sinnott & Otto 2009, Goggs *et al*. 2012). The lack of correlation between CTPA results and outcome in these patients is noteworthy and is most likely explained by the low case numbers. The argument for definitively diagnosing PTE with CTPA is to enable administration of specific treatment to dogs with thromboembolic disease, such as antithrombotic therapies or supportive medications including sildenafil, which may improve outcome. Definitive PTE diagnosis is a requisite for interventional clinical trials, which in humans have identified potentially beneficial interventions for PTE such as rivaroxaban and low-dose thrombolysis (Büller *et al*. 2012, Sharifi *et al*. 2013). Such studies in dogs are not currently available, but might be possible once PTE can be routinely diagnosed.

It is recognised that this study has limitations. This investigation was designed as a pilot study, with a small planned enrollment, but clearly represents a small sample of the dogs treated at the institution. All dogs in this study were deemed high-risk for PTE. Although this increased the pretest probability of PTE, it did enable evaluation of the feasibility of CTPA for PTE diagnosis in dogs. The study was also limited by the lack of a gold-standard against which to compare CTPA. V/Q scintigraphy and selective pulmonary angiography have previously been used for PTE diagnosis in dogs (Suter 1984, Bunch *et al*. 1989, Johnson *et al*. 1999), but V/Q scanning was not available and selective pulmonary angiography is an invasive and potentially high-risk procedure in unstable cases.

This study was undertaken prospectively to maximise the completeness of data collection. Despite this precaution, some data are missing. In two cases the omission of data was due to deterioration in the dog's condition. The missing data limit the ability to evaluate the predictive ability of non-imaging diagnostic tests for the presence of PTE; however, this study does provide a clear basis for future, larger studies in this area.

In summary, the feasibility of CTPA for identification of naturally occurring PTE in dogs has been established and that CTPA can be successfully performed under sedation, even in cases with respiratory distress, has been demonstrated. This study also shows that CTPA can both confirm and rule out PTE in major pulmonary arteries in these cases. Although few of the other diagnostic tests for PTE correlated with CTPA in this study, larger studies of this and other populations can now be undertaken using the protocols established here to more fully assess their diagnostic potential.

## SUPPORTING INFORMATION

The following supporting information is available for this article:

Table S1. Summary historical, physical examination, diagnostic, clinicopathologic and treatment data for the 12 dogs with IMHA included in this study.
